# Baseline Prevalence of Birth Defects Associated with Congenital Zika Virus Infection — Massachusetts, North Carolina, and Atlanta, Georgia, 2013–2014

**DOI:** 10.15585/mmwr.mm6608a4

**Published:** 2017-03-03

**Authors:** Janet D. Cragan, Cara T. Mai, Emily E. Petersen, Rebecca F. Liberman, Nina E. Forestieri, Alissa C. Stevens, Augustina Delaney, April L. Dawson, Sascha R. Ellington, Carrie K. Shapiro-Mendoza, Julie E. Dunn, Cathleen A. Higgins, Robert E. Meyer, Tonya Williams, Kara N.D. Polen, Kim Newsome, Megan Reynolds, Jennifer Isenburg, Suzanne M. Gilboa, Dana M. Meaney-Delman, Cynthia A. Moore, Coleen A. Boyle, Margaret A. Honein

**Affiliations:** ^1^Division of Congenital and Developmental Disorders, National Center on Birth Defects and Developmental Disabilities, CDC; ^2^Division of Reproductive Health, National Center for Chronic Disease Prevention and Health Promotion, CDC; ^3^Center for Birth Defects Research and Prevention, Massachusetts Department of Public Health; ^4^Birth Defects Monitoring Program, North Carolina Department of Health and Human Services; ^5^Division of Human Development and Disability, National Center on Birth Defects and Developmental Disabilities, CDC; ^6^Office of the Director, National Center for Emerging and Zoonotic Diseases, CDC; ^7^Office of the Director, National Center on Birth Defects and Developmental Disabilities, CDC.

Zika virus infection during pregnancy can cause serious brain abnormalities, but the full range of adverse outcomes is unknown (*1*). To better understand the impact of birth defects resulting from Zika virus infection, the CDC surveillance case definition established in 2016 for birth defects potentially related to Zika virus infection* (*2*) was retrospectively applied to population-based birth defects surveillance data collected during 2013–2014 in three areas before the introduction of Zika virus (the pre-Zika years) into the World Health Organization’s Region of the Americas (Americas) (*3*). These data, from Massachusetts (2013), North Carolina (2013), and Atlanta, Georgia (2013–2014), included 747 infants and fetuses with one or more of the birth defects meeting the case definition (pre-Zika prevalence = 2.86 per 1,000 live births). Brain abnormalities or microcephaly were the most frequently recorded (1.50 per 1,000), followed by neural tube defects and other early brain malformations^†^ (0.88), eye abnormalities without mention of a brain abnormality (0.31), and other consequences of central nervous system (CNS) dysfunction without mention of brain or eye abnormalities (0.17). During January 15–September 22, 2016, the U.S. Zika Pregnancy Registry (USZPR) reported 26 infants and fetuses with these same defects among 442 completed pregnancies (58.8 per 1,000) born to mothers with laboratory evidence of possible Zika virus infection during pregnancy (*2*). Although the ascertainment methods differed, this finding was approximately 20 times higher than the proportion of one or more of the same birth defects among pregnancies during the pre-Zika years. These data demonstrate the importance of population-based surveillance for interpreting data about birth defects potentially related to Zika virus infection.

Statewide data from birth defects surveillance programs in Massachusetts and North Carolina for 2013 and from a surveillance program in three counties in metropolitan Atlanta, Georgia, for 2013–2014 were chosen for analysis because these programs conducted population-based surveillance for all types of birth defects, used active multisource case-finding, and were rapidly able to provide individual-level data with sufficient detail to apply all inclusion and exclusion criteria (*4*). Trained staff members in these surveillance programs routinely reviewed the medical records of infants and fetuses with birth defects and abstracted information about those defects, related diagnostic procedures, and demographic and pregnancy information. Included were all infants and fetuses who were identified through surveillance with a birth defect characterized by CDC subject matter experts as being consistent with those observed in cases of congenital Zika virus infection (*2*). Additional data collected included the pregnancy outcome (live birth or pregnancy loss), maternal age, gestational age at delivery, and verbatim clinical descriptions of all birth defects, including genetic abnormalities. These verbatim descriptions were reviewed by CDC subject matter experts to verify the case definition and categorization. The earliest age that a birth defect meeting the definition was noted (i.e., prenatally, ≤28 days after delivery, 29 days to <3 months after delivery, ≥3 to <6 months after delivery, and ≥6 months after delivery) was available for data from Massachusetts and Atlanta.

Infants or fetuses with birth defects were aggregated into four mutually exclusive categories of defects characterized by CDC subject matter experts as being consistent with those observed with congenital Zika virus infection: 1) brain abnormalities or microcephaly (head circumference at delivery <3rd percentile for sex and gestational age) (*5*); 2) neural tube defects and other early brain malformations; 3) eye abnormalities without mention of a brain abnormality included in the first two categories; and 4) other consequences of CNS dysfunction, specifically joint contractures and congenital sensorineural deafness, without mention of brain or eye abnormalities included in another category. Baseline prevalence per 1,000 live births (*6*) and 95% confidence intervals (CIs) were estimated using Poisson regression.

The three birth defects surveillance programs identified 747 infants and fetuses during 2013 (North Carolina and Massachusetts) and 2013–2014 (Atlanta) with one or more defects that met the 2016 CDC Zika surveillance case definition (2.86 per 1,000 live births [CI = 2.65–3.07]) (Table). Brain abnormalities or microcephaly accounted for the largest number (392 [52%]) and highest prevalence (1.50 per 1,000), followed by neural tube defects and other early brain malformations (229 [31%]; 0.88). Eye abnormalities without mention of a brain abnormality (81 [11%]; 0.31) and consequences of CNS dysfunction without mention of brain or eye abnormalities (45 [6%]; 0.17) were less frequent. Pregnancy losses (48%) and preterm delivery (<37 weeks’ gestation) (66%) occurred most frequently with neural tube defects and other early brain malformations. In contrast, all infants with eye abnormalities without mention of a brain abnormality were liveborn.

**TABLE:** Reports of birth defects potentially related to congenital Zika virus infection* collected during a pre-Zika period, by selected characteristics — Massachusetts, North Carolina, and Atlanta, Georgia, 2013–2014^†^

Characteristic	Brain abnormalities or microcephaly (%)	NTDs and other early brain malformations (%)	Eye abnormalities (%)	Other consequences of CNS dysfunction (%)	Total
**No. of infants or fetuses (N = 747)**	392 (100)	229 (100)	81 (100)	45 (100)	**747**
**Pregnancy outcome**					
Live birth	349 (89)	119 (52)	81 (100)	43 (96)	**592**
Pregnancy loss^§^	43 (11)	109 (48)	0 (—)	2 (4)	**154**
**Gestational age at delivery (wks)**					
<32	68 (17)	114 (50)	6 (8)	7 (16)	**195**
32–36	80 (20)	37 (16)	18 (22)	9 (20)	**144**
37–41	243 (62)	76 (33)	56 (69)	29 (64)	**404**
≥42	1 (<1)	2 (1)	1 (1)	0 (—)	**4**
**Maternal age at delivery (yrs)**					
<25	127 (32)	49 (22)	15 (18)	15 (33)	**206**
25–34	178 (45)	122 (54)	42 (52)	21 (47)	**363**
≥35	87 (22)	56 (25)	24 (30)	9 (20)	**176**
**Earliest age birth defect was noted (n = 410)^¶^**					
Prenatally	116 (55)	104 (89)	4 (7)	5 (18)	**229**
≤28 days of delivery	58 (27)	9 (8)	29 (54)	19 (70)	**115**
29 days to <3 months	13 (6)	3 (3)	10 (18)	1 (4)	**27**
3 months to <6 months	10 (5)	1 (1)	3 (6)	2 (7)	**16**
≥6 months	15 (7)	0 (—)	8 (15)	0 (—)	**23**
**Fetuses/Infants with defects per 1,000 live births (95% CI)**	1.50 (1.35–1.65)	0.88 (0.77–1.00)	0.31 (0.25–0.38)	0.17 (0.13–0.23)	**2.86 (2.65–3.07)**

**Abbreviations:** CNS = central nervous system; CI = confidence interval; NTD = neural tube defect.

* Case reports were aggregated into four mutually exclusive defect categories: 1) brain abnormalities or microcephaly (defined as head circumference at delivery <3rd percentile for sex and gestational age); 2) NTDs and other early brain malformations (these are included as biologically plausible but have been reported much less frequently with Zika virus infection than those in category 1); 3) eye abnormalities (without mention of a brain abnormality in categories 1 or 2); and 4) other consequences of CNS dysfunction, specifically joint contractures and congenital sensorineural deafness, without mention of brain or eye abnormalities included in any other category.

^†^ Data from Massachusetts (2013), North Carolina (2013), and three counties in metropolitan Atlanta, Georgia, (2013–2014). Total live birth population for the three areas = 261,629.

^§^ Includes stillbirths ≥20 weeks gestation, elective terminations after prenatal diagnosis at any gestational age and, in Massachusetts, spontaneous pregnancy losses at <20 weeks and <350 g.

^¶^ The earliest age when a birth defect meeting the 2016 CDC Zika surveillance case definition was first noted in the medical record was only available for 410 cases from Massachusetts and metropolitan Atlanta, Georgia.

In general, the distribution by maternal age was similar across birth defect categories. Among 410 (55%) infants or fetuses with information on the earliest age a birth defect was recorded, 371 (90%) had evidence of a birth defect meeting the Zika definition before age 3 months. More than half of those with brain abnormalities or microcephaly or with neural tube defects and other early brain malformations had evidence of these defects noted prenatally (55% and 89%, respectively).

## Discussion

A congenital Zika syndrome phenotype has been described (*7*); however, the birth defects observed are not unique to congenital Zika virus infection, and the full range of effects of congenital Zika infection is not known. The data in this report provide a baseline reference for the prevalence of defects observed with congenital Zika virus infection in the pre-Zika years and demonstrate the importance of data on birth defects prevalence in providing a context within which to assess the impact of teratogenic exposures such as Zika virus infection. Recently published data from the USZPR reported 26 infants and fetuses with these same birth defects among 442 completed pregnancies with laboratory evidence of Zika virus infection during a 9-month period in 2016. This proportion (58.8 per 1,000) is approximately 20 times higher than the prevalence (2.86 per 1,000) from the three population-based birth defects surveillance programs during the pre-Zika years. In addition, of the 26 USZPR infants and fetuses, 22 had a brain abnormality or microcephaly (*2*). This proportion (49.8 per 1,000; CI = 33.1–74.8) is approximately 33 times higher than the prevalence (1.5 per 1,000) among pregnancies in the pre-Zika years.

A recently published report from New York took a somewhat different approach to establishing a pre-Zika baseline for congenital birth defects. It examined diagnoses of microcephaly, but not other defects, for the period 2013–2015 and found that, before evidence of importation of Zika virus infections, the overall prevalence of microcephaly in New York was 7.4 per 10,000 live births (0.74 per 1,000), and the prevalence of severe congenital microcephaly (newborn head circumference <3rd percentile for sex and gestational age) was 4.2 per 10,000 (0.42 per 1,000) (*8*).

The findings in this report are subject to at least six limitations. First, population-based surveillance programs strive to ascertain the prevalence of birth defects among all members of a specified population. In contrast, the aim of USZPR is to estimate the proportion of birth defects among pregnancies with laboratory evidence of possible Zika virus infection, a specific subgroup of the general population (*2*). This could lead to selection bias with USZPR if, for example, pregnancies with fetal abnormalities detected prenatally were more likely to be tested for Zika virus and reported. Second, birth defects surveillance programs identify diagnoses among infants and fetuses mostly through review of administrative records, often at inpatient facilities. Although these programs use multisource ascertainment, some birth defects could be missed if they were prenatally diagnosed or if infants were delivered at sites outside of the usual ascertainment sources, if infants were evaluated solely in outpatient settings, or if some birth defect diagnoses did not receive an administrative code. In contrast, USZPR receives reports of pregnant women with laboratory evidence of possible Zika virus infection and resulting fetal and infant outcomes. The prospective nature of this ascertainment and direct follow-up of individual reported pregnancies could result in closer scrutiny of the outcomes and more frequent and detailed detection of abnormalities than is typical with population-based birth defects surveillance programs. Third, data from these three birth defects surveillance programs might not be generalizable to the United States. The USZPR-published data included reports from any of the U.S states and the District of Columbia. Also, it is possible that some pregnancies with Zika virus infection were present in the birth defects surveillance populations during the pre-Zika years as a result of travel to areas with Zika virus outside the Americas. Fourth, birth defects surveillance programs traditionally do not ascertain diagnoses from settings where congenital deafness is diagnosed; therefore, these data likely do not include the majority of infants with congenital sensorineural deafness. Fifth, published data from USZPR on the proportion of infants and fetuses with other types of birth defects that are not thought to result from congenital Zika virus infection are not available, making it impossible to assess differences in the frequency of other birth defects. Finally, published data from USZPR include many pregnancies with unspecified flavivirus infections, and thus the estimates of the proportion with birth defects potentially related to Zika virus infection might underestimate the actual Zika impact, given that some included pregnant women likely had other flavivirus infections, increasing the size of the denominator.

The birth defects surveillance data in this report were compiled from a period before introduction of Zika virus in the Americas, using the CDC surveillance case definition of birth defects potentially related to Zika virus infection; this is the same case definition adopted by USZPR. The higher proportion of these defects among pregnancies with laboratory evidence of Zika infection in USZPR supports the relationship between congenital Zika virus infection and these birth defects (*1*,*2*). These data demonstrate the critical contribution of population-based birth defects surveillance to understanding the impact of Zika virus infection during pregnancy. In 2016, CDC provided funding for 45 local, state, and territorial health departments to conduct rapid population-based surveillance for defects potentially related to Zika virus infection, which will provide essential data to monitor the impact of Zika virus infection in the United States.

**Summary**

**What is already known about this topic?**

Zika virus infection causes serious brain abnormalities; however, the birth defects observed are not unique to congenital Zika virus infection, and the full range of effects of congenital Zika infection is not known.

**What is added by this report?**

CDC used data from population-based birth defects surveillance programs in Massachusetts, North Carolina, and Atlanta, Georgia, to retrospectively assess the prevalence of birth defects during 2013–2014 that met the surveillance case definition for birth defects potentially related to Zika virus infection, before introduction of Zika virus into the United States. After introduction of Zika virus, the proportion of infants and fetuses with birth defects born to mothers with laboratory evidence of possible Zika infection reported by the US Zika Pregnancy Registry during January 15–September 22, 2016, was approximately 20 times higher than the prevalence of potentially Zika-related birth defects among pregnancies during the pre-Zika years.

**What are the implications for public health practice?**

Data on birth defects in the pre-Zika years serve as benchmarks to direct rapid ascertainment and reporting of birth defects potentially related to Zika virus infection. The higher proportion of these defects among pregnancies with laboratory evidence of possible Zika virus infection supports the relationship between congenital Zika virus infection and birth defects.

## Footnotes

* The Zika surveillance case definition covers all birth defects that have been reported as potentially related to Zika virus infection and includes brain abnormalities such as microcephaly, intracranial calcifications, fetal brain disruption sequence, abnormal cortical formation, and porencephaly, among others; neural tube defects and other early brain malformations, such as anencephaly, spina bifida, encephalocele, and holoprosencephaly; eye abnormalities, such as microphthalmia/anophthalmia, cataracts, chorioretinal and optic nerve abnormalities, among others; and consequences of central nervous system dysfunction, such as joint contractures and congenital sensorineural deafness.

† Neural tube defects and other early brain malformations are included as biologically plausible birth defects; however, they have been reported much less frequently with Zika virus infection than defects in the other categories.
